# Tuberculosis in Solomon Islands: why declining case notifications may not reflect true incidence

**DOI:** 10.5365/wpsar.2024.15.3.1174

**Published:** 2024-09-17

**Authors:** Dylan M Bush, Alice Siuna Waneoroa, Emire Meone Maefiti, Thomas H Fitzpatrick, Elizabeth Wore, Silvia S Chiang

In a recent publication, Yanagawa et al. report that the decrease in tuberculosis (TB) case notifications between 2016 and 2022 in Solomon Islands represents a “major achievement” and may indicate an “actual reduction in TB incidence.” (*1*) We offer additional context to highlight major limitations in TB detection in Solomon Islands and advise caution in the interpretation of this result.

TB detection in Solomon Islands is difficult due to geographic and resource limitations. Solomon Islands is a Western Pacific nation consisting of nearly 1000 islands. Paved roads are almost non-existent outside of the capital, Honiara, which is home to the country’s only tertiary care centre. Although roughly 80% of Solomon Islanders live in rural areas, approximately 75% of doctors are based in the capital. (*2*) Prior research has highlighted underreporting of TB cases and high rates of TB treatment failure in rural areas, (*3*) where people face logistical and cultural barriers to medical care. (*4*) Current diagnostic tools for TB in Solomon Islands include Xpert® MTB/RIF Ultra, which was introduced in 2023, sputum smear microscopy and chest X-ray. However, Xpert® MTB/RIF testing and X-ray are not available in all provinces.

Furthermore, in 2021, community-based contact tracing ceased due to workforce constraints. Due to disruptions related to the COVID-19 pandemic, contact tracing was only carried out for immediate family members who presented with the patient to the medical facility. Thus, other contacts – including children – who did not present with the patient to the facility were not evaluated. This change may have contributed to decreased case notifications in 2022. Although community-based contact tracing resumed in 2023, contacts are evaluated only once within 2 weeks of the patient’s presentation to a hospital. Given the potential delay of up to 12 weeks for tests of TB infection to turn positive, it is important to repeat testing.

Some findings reported by Yanagawa et al. suggest underdiagnosis of TB disease. The authors note a higher case notification rate among adult women than men. However, in most endemic settings, TB incidence is substantially higher among males. (*5*) As noted by Yanagawa et al., few members of high-risk populations, such as those with diabetes mellitus, were screened for TB. (*1*) Taken together with the 67% screening positivity in this group and the high incidence of diabetes mellitus in Solomon Islands, this finding suggests a high likelihood of TB underdetection in this population.

It is notable that the authors suggest that TB incidence may have decreased in Solomon Islands in recent years, despite most other countries reporting increased TB incidence following the pandemic due to reduced detection and increased community spread. (*6*) As previously mentioned, contact tracing was suspended during the pandemic in Solomon Islands. Moreover, in this country, where protocols mandate hospitalization during the intensive phase of TB treatment, the number of beds in TB units was reduced as they were reallocated to patients with suspected COVID-19. Consequently, patients with TB often remained in general medical or emergency wards, likely leading to increased nosocomial transmission.

The authors point to the “sustained population testing rate” over the surveillance period in combination with reduced case notifications as supportive of a reduction in TB incidence. However, according to the authors, only 0.13–0.18% of the population was evaluated each year. (*1*) This very small proportion is unlikely to be representative of the general population.

In summary, Yanagawa et al. have published an important paper on TB in Solomon Islands. Yet, given the high likelihood of TB underdetection, we advise caution in interpreting the decrease in TB notifications as reflecting a true reduction in incidence. We also urge further investment and research, which would strengthen active case-finding, more accurately capture TB incidence in Solomon Islands, improve effective TB diagnosis and treatment and reduce community transmission.
